# Eosinophilic granuloma affecting the parietal bone of the skull: A case report and literature review

**DOI:** 10.1016/j.ijscr.2022.107371

**Published:** 2022-06-29

**Authors:** Osama N. Dukmak, Sulaiman M.S. Abualia, Yara J.I. Meqbil, Mohammad Emar, Sharif Issa Basal, Saeed Itaidek

**Affiliations:** a Al-Quds University - Faculty of Medicine, Jerusalem, State of Palestine; b Al-Ahli Hospital, Hebron, State of Palestine

**Keywords:** Eosinophilic granuloma, Langerhans cell histiocytosis, Parietal bone, Bony lytic lesion, Offensive odor, Case report

## Abstract

**Background:**

The rare form and mildest variant of Langerhans cell histiocytosis is eosinophilic granuloma (EG). In the clinical presentation, EG can be monostotic, polyostotic, or can encompass many organs. The parietal bone is the most common location of the skull bones that are affected by EG. So far, there have been no reported cases of EG with skull odor as an unexplained presentation.

**Case presentation:**

An 8-year-old girl presented with a 4 months history of a right parietal bone swelling of the skull with an offensive odor. There was no discharge and no history of vomiting or trauma. An MRI scan of the brain showed swelling with a bone lesion of the right parietal bone. Infection was the source of the swelling and the bad odor. Treatment was done by surgical excision of the lesion.

**Conclusion:**

EG has a variety of presentations and should be suspected when tenderness and local swelling are present. Radiography was found to be helpful in the diagnosis and surgical treatment was done to manage the case.

## Highlights

• Most common site to be affected by EG in the Skull is the parietal bone.
• EG may be infected and present with an offensive odor.
• EG cells Stains positive for S-100 and CD1a.
• Most of the Cases are reported among children and most of them present with Headache.

## 1 Introduction

Langerhans cell histiocytosis (LCH) is an idiopathic disorders group characterized by disordered histiocytes proliferation. LCH is rare, but it is more common in children than in adults [1]. Unifocal LCH is the modern name for eosinophilic granuloma. Eosinophilic granuloma is an uncommon subtype of LCH and it is the mildest form of it [2]. The progression of the disease is slow and is characterized by the expansion of Langerhans cells, which usually affect the bones of the skull as a destructive lesion [1]. We report here an extremely rare case of eosinophilic granuloma affecting the parietal bone of the skull in an 8-year-old girl presenting with an offensive head odor.

This case has been reported in line with SCARE criteria which is mentioned in Methods section.

## 2 Case presentation

**Fig. 1:**
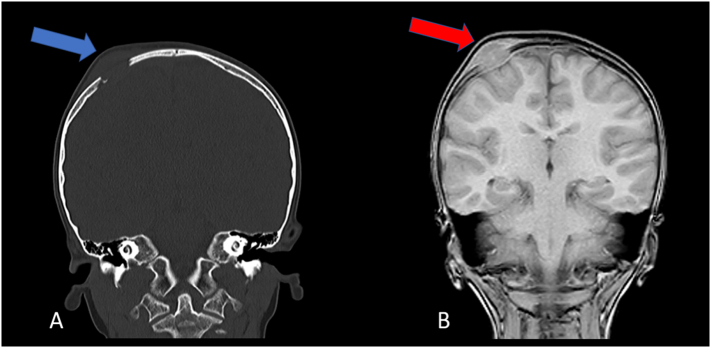
(A) CT scan shows a lytic bony lesion (Blue Arrow). (B) MRI scan shows a 2.5 × 1.5 cm bone lesion extended as swelling (Red Arrow). (For interpretation of the references to colour in this figure legend, the reader is referred to the web version of this article.)

**Fig. 2:**
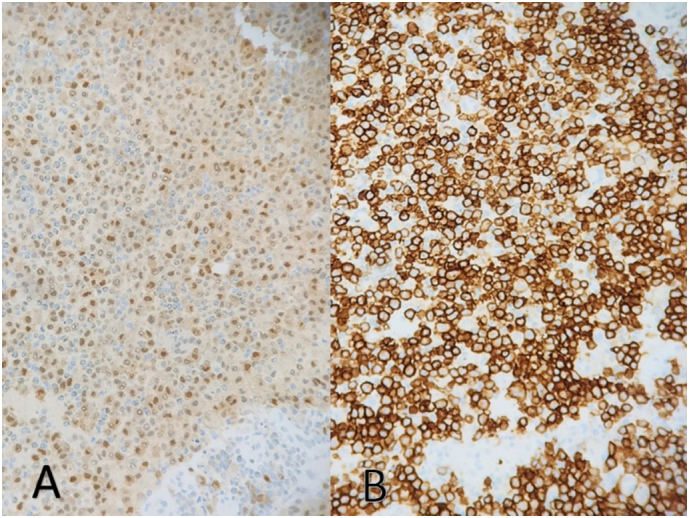
(A) Shows that the lesion stained positive for S100. (B) Shows that the lesion stained positive for CD1a.

**Fig. 3:**
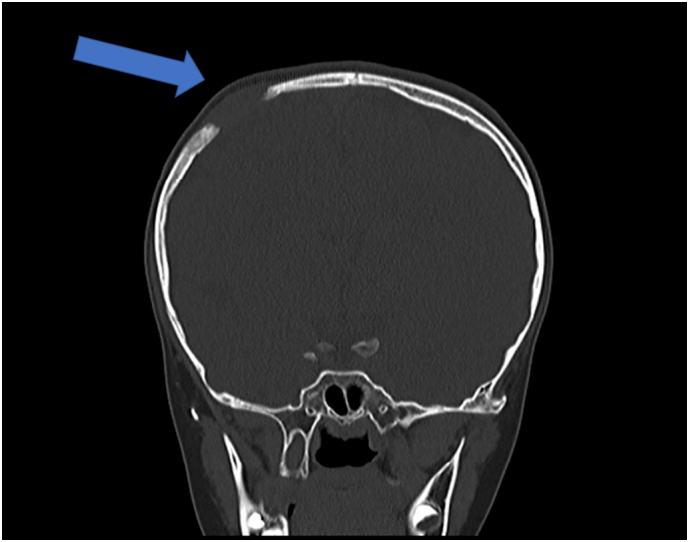
CT scan shows a lytic lesion after treating the infection (Blue Arrow). (For interpretation of the references to colour in this figure legend, the reader is referred to the web version of this article.)

**Fig. 4:**
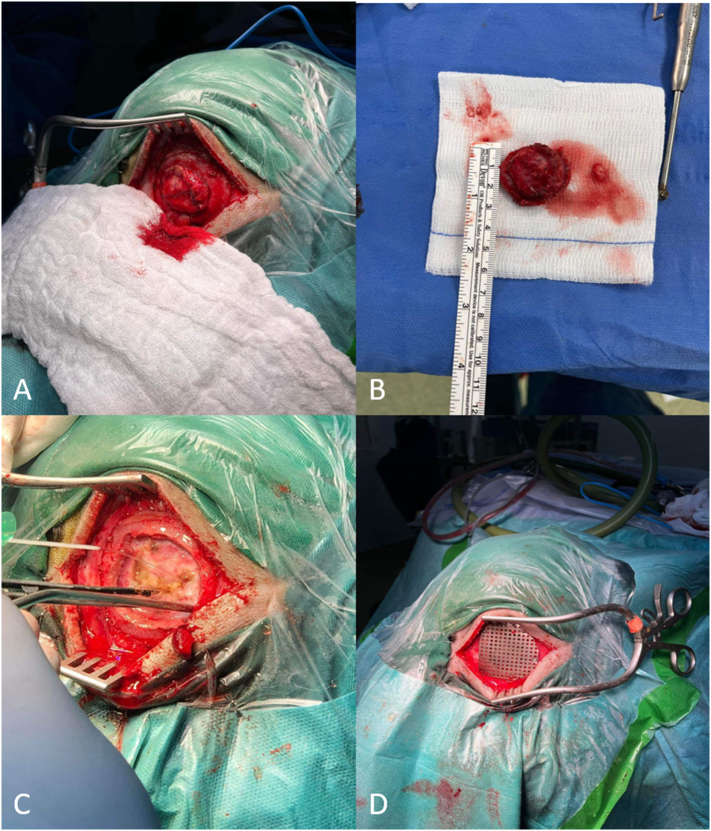
(A and B) Show the 2.5 × 1.5 cm bone lesion. (C) The excision of the lesion with a 3 mm safety margin. (D) The insertion of titanium mesh.

An 8-year-old girl presented with a 4 months history of right parietal bone swelling with an offensive odor. There was no discharge and no history of vomiting or trauma. She has taken no medications lately and had no allergic reactions. In the clinic, she had a mild headache with no fever, no symptoms of increased intracranial pressure, and no neurological abnormalities. A head computed tomography (CT) scan showed a parietal bone lesion. After that, an MRI scan of the brain showed a 2.5 × 1.5 cm bone lesion extended as swelling and suggested the diagnosis of EG (Fig. 1). Histopathology was performed and revealed proliferation of monotonous population of discohesive cells composed of oval nuclei with open chromatin and coffee bean grooves with abundant pale cytoplasm. Immunohistochemistry for these cells was positive for CD1a and S100 (Fig. 2). Initial treatment with augmentin was performed to relieve the infection and the odor. After that, a CT scan showed that the swelling is disappeared, but there was a lytic bone lesion (Fig. 3). On examination, there was a feeling of soft tissue. Thereupon, a surgical procedure was done by excision of the lesion with a 3 mm safety margin, and a titanium mesh was inserted (Fig. 4). During the surgery, the patient was given cefazolin. Laboratory values before and after the surgery were within the normal range. The patient was discharged home with augmentin and analgesia. In the clinic, the follow-up of the patient was performed with no post-operative complications.

## 3 Discussion

Eosinophilic granuloma (EG) is one type of Langerhans cell histiocytosis (LCH) that involves any bone. The most frequent sites of EG vary with age. The skull (40 %), rib, femur, humerus, and vertebra are the most common sites in children [3]. EG is a rare tumor-like lesion that represents less than 1 % of all bone lesions [4]. Tenderness and local swelling are usually the presenting symptoms [5]. Differential diagnoses of EG include dermoid cysts and sebaceous cysts, osteoblastomas, hemangiomas, and osteogenic sarcomas [6].

The presence of eosinophilic granuloma may be indicated by its location, symptoms, or radiologic appearance. The evaluation of EG includes radiographic tests, laboratory tests, and histopathology to confirm a diagnosis [7]. In our case, the initial concept that came to our minds was an infected sebaceous cyst. But on physical examination, the case was not correlated with sebaceous cyst. Laboratory tests have been done and the results were within the normal range. Thus, this finding ruled out osteomyelitis and other malignancies. We proceeded to the CT scan and eosinophilic granuloma was suspected. Computed tomographic scan and magnetic resonance imaging may detect punched-out lesions as well as soft tissue involvement adjacent to the bony structure [7]. Accordingly, CT and MRI were done. Moreover, tumor cells have to stain positively for CD1a and S100 on immunohistochemistry to be identified as Langerhans cells [8]. As a result, a biopsy was taken and the case was diagnosed.

The pathophysiology of eosinophilic granuloma is unclear. However, the proliferation of Langerhans cells may be induced by viral infection, immune dysfunction, or bacterial infection. As a result, interleukin-1 and interleukin-10 may be elevated [9]. Therefore, antibiotics could be used to relieve the bacterial infection and the swelling that results from it [7].

Treatment and prognosis for eosinophilic granuloma of the skull depend on age and number of bones involved at the time of diagnosis [10]. In terms of treatment, it is divided into two groups, non-operative and operative. The non-operative methods include observation; solitary lesions usually resolve spontaneously, irradiation and chemotherapy [7]. As for the operative treatment, surgical excision and grafting are performed. In our case, operative treatment was done and grafting was performed by inserting a titanium mesh.

After reviewing the literature, there was only one case with EG that presented with an offensive odor. In that case, the mandible was the affected site [11]. The well-defined lytic lesion of eosinophilic granuloma in the skull is most commonly seen in the parietal and frontal bones [12]. EG frequently occurs in children as seen in Table 1. Most of the cases in the table were presented with headache which varied in severity. Some cases presented with other symptoms such as nausea and vomiting. On the other hand, the minority of the cases were found to have neurological symptoms such as seizures. 4 cases were reported with extradural hematoma, and one of them had a history of trauma [13]. Noteworthy, there is a remarkable presentation that has been reported in our case which is the offensive odor in the parietal bone.

**Table 1:** Summary of unusual findings in reported cases of eosinophilic granuloma affecting the parietal bone as reported in the literature.

Year	Gender	Age/years	Presentation				Radiographic findings
			Mass	Duration	Trauma	Symptoms	
1970 [14]	Female	38	Tender	3 months	No	Seizure activity on EEG	Large irregular osteolytic defect
1973 [15]	Male	5	Smooth, non-tender	–	No	None	Circular localized area of destruction with clear cut margins
1990 [16]	Male	2	Soft and tender with smooth surfaces	1 month	No	None	Soft tissue masses with destruction in the frontal and right parietal areas
1990 [16]	Male	2	Soft, painless	1 month	No	None	Punched-out and well-defined lytic lesion
2006 [6]	Female	26	Tender and firm	2 weeks	No	Progressive headache and nausea	Hyperintense osteolytic lesion
2007 [17]	Male	32	Edematous	–	Yes/3 years	Headache	Hypointense lesion with perilesional edema
2007 [18]	Male	37	Soft tissue	2 months	No	Headache and epileptic attacks	Osteolytic lesion with a large epidural and subcutaneous mass
2009 [19]	Female	36	Tender	2 months	No	Headache	Single punched out area of bone destruction with sharp margins
2010 [20]	Male	10	Tender	1 months	No	None	Lytic bone lesion extended as swelling with extradural hematoma
2011 [21]	Male	4	Large and gradually decreasing in size	1 month	Yes/1 month	Unremitting headache and projectile vomiting	Lytic lesion, scalp swelling and hematoma
2013 [22]	Female	14	Tender and soft	1 month	No	Headache, malaise and nausea	Osteolytic change with extracranial swelling
2013 [23]	Male	44	Immobile palpable masses	–	No	Multiple cranial swellings accompanied by pain	Radiolucent areas in the right frontoparietal, parietal and temporal bones
2016 [24]	Male	7	Non-tender	2 months	No	Progressive headache, decreased level of consciousness and vomiting	Heterogeneous osteolytic mass with extradural hematoma
2018 [25]	Female	8	No mass was felt	6 weeks	No	Focal pain at the scalp	Large osteolytic defect with non-sclerotic margins and beveled edges.
2019 [26]	Female	10	Tender	3 weeks	Yes/2 weeks	None	Single osteolytic lesion without sclerosis
2020 [13]	Male	3	Non-tender	2 days	No	Recurrent and progressive vomiting and drowsiness	Iso-dense subcutaneous scalp lesion, underlying osteolytic bony defect and mixed density extradural lesion with extradural hematoma
2021 [27]	Female	18	Tender	3 weeks	No	Headache	Lytic lesion with disruption of the external tabula and an epicranial soft tissue extension of the lesion
2021 [28]	Male	10	Non-tender and gradually increasing in size	–	Yes	Pain in the local area of the swelling	3 cm sized defect in the right temporoparietal calvarial with scalloped margin
2022	Female	8	Non-Tender	4 months	No	Offensive odor with mild headache	Lytic bone lesion extended as swelling

## 4 Conclusion

We present here a case report of an extremely rare eosinophilic granuloma in the parietal bone of a pediatric patient. Noteworthy, EG should be suspected when tenderness and local swelling are present. Our case was presented with an offensive odor with no clear reason. After reviewing the literature, we did not detect any cases to have a similar finding. In our case, radiography was found to be helpful in the diagnosis. Furthermore, surgical treatment was done to manage the case.

## Methods

This case has been reported in line with SCARE Criteria [29].

## Source of funding

The study did not receive any funding.

## Ethical approval

This study is exempt from ethical approval at our hospital.

## Consent

Written informed consent was obtained from the patient for reporting this case. The consent is available for review on request.

## Authors' contributions

**Data collection**: Osama N. Dukmak, Mohammad Emar.

**Writing the manuscript**: Osama N. Dukmak, Sulaiman M. S. Abualia, Yara J. I. Meqbil.

**Study concept or design**: Osama N. Dukmak, Sulaiman M. S. Abualia, Yara J. I. Meqbil.

**Review & editing the manuscript**: Osama N. Dukmak, Yara J. I. Meqbil, Sulaiman M. S. Abualia, Mohammad Emar.

## Registration of research studies

N/A.

## Guarantor

Dr. Saeed Idkedek.

## Provenance and peer review

Not commissioned, externally peer-reviewed.

## Declaration of competing interest

There is no conflict of interest to declare.
